# Can AI Robot Teaching Improve Children’s Performance in Housework? A Quasi-Experimental Study

**DOI:** 10.3390/children11111330

**Published:** 2024-10-30

**Authors:** Ching-Fen Lee, Shain-May Tang

**Affiliations:** 1 Department of Early Childhood Education and Care, Minghsin University of Science and Technology, Hsinchu County 304001, Taiwan; 062888@yahoo.com.tw; 2 Department of Living Science, National Open University, New Taipei City 247340, Taiwan

**Keywords:** AI robots, children, housework performance, quasi-experimental study

## Abstract

Background: The primary objective of this study is to explore the effects of using Artificial Intelligence (AI) robot-assisted instruction in preschool activities on young children’s performance in housework. Methods: A quasi-experimental design was employed, with an experimental group and two control groups, to observe changes in children’s performance in housework before and after AI robot-assisted teaching. The study sample consisted of preschoolers from metropolitan Taipei, with 65 children in the experimental group, 53 in control group 1, and 75 in control group 2. Children participating in the research project required consent from the school, teachers, and parents. The children in the experimental group received AI robot-assisted instruction on household tasks for two days a week over three consecutive weeks, with each session lasting approximately 20–30 min. Control group 1 did not receive any experimental treatment, while control group 2 underwent the same learning schedule and content as the experimental group but was instructed by teachers instead of AI robots. The data were analyzed based on pre- and post-test surveys of parents observing their children’s performance in household tasks. Results: This study found that both AI robot-assisted teaching and teacher-led instruction enhanced children’s household skills, with the AI robot-assisted method showing slightly better results. These findings suggest that both technological tools and teacher guidance can effectively improve children’s housework performance, while artificial intelligence robots may provide young children with motivation and curiosity to learn due to their appearance and interactive design, which deserves further analysis. Conclusion: As technology rapidly advances, young children are exposed to various technological devices at an early age. Integrating technological media into future preschool teaching is inevitable, and leveraging tools like AI robots to support teaching, reduce teacher burden, and diversify instructional methods could be a direction worth considering.

## 1. Introduction

### 1.1. The Importance of Children’s Participation in Housework

Many studies on children’s participation in housework have focused on children of school age or above and are based on developmental theory and socialization perspectives [1]. Preschool children’s involvement in chores is often overlooked as their contributions are viewed as unable to provide substantial help with household tasks [2], and may even increase parents’ workload during the process [3]. Moreover, in Chinese societies, the cultural emphasis on academic achievement over housework training results in fewer opportunities for children to learn chores [4], which indirectly reduces attention to this issue.

Although there is still debate about whether young children can effectively contribute to household chores, it is undeniable that appropriate participation in chores benefits their growth and overall development. When children engage in activities like tidying up toys, assisting with laundry, household maintenance, or gardening, their physical activity increases [5,6], which can reduce sedentary behavior and benefit their health [7]. Moreover, appropriate participation in household tasks helps in the development of language as well as physical, cognitive, social, intellectual, independence, and executive skills, with these benefits persisting into the future [7,8]. Participation in chores also nurtures a sense of family and social responsibility, enhances socialization [5,9], reduces parent–child conflicts [10], improves parent–child interactions [11], and lowers parental stress [12], thereby enhancing children’s overall life satisfaction [13].

### 1.2. Household Tasks for Young Children

Household tasks are necessary to maintain daily family life and are not limited to daily chores; they span the physical, mental, emotional, and spiritual dimensions [14]. For young children, housework is also closely related to their personal and family daily activities but it is more related to their developmental abilities because the difficulties of many household tasks are beyond their capabilities. Young children’s performance in housework and projects will change as they grow.

Goodnow [15] and Dunn [16] categorized children’s chores into self-care tasks, which involve tasks related to the child’s own care or managing their personal space, such as making their own bed; and family care tasks, which involve caring for family members and the household in shared living spaces, such as washing dishes.

However, unlike Goodnow’s study, Dunn’s Chores Scale measures children’s ability to perform chores—rather than how long or often they participate in chores—because children with disabilities often experience developmental delays and often cannot complete these household tasks independently. Dunn’s scale design is also very suitable for preschoolers who are unable to complete household chores due to immature development. Therefore, this study will refer to Dunn’s measurement and focus on analyzing children’s housework abilities.

The content of household chores for preschoolers, as suggested in the study by Lee and Tang [17], is more suitable for the participants in this research. Also, we do not analyze self-care tasks because the majority of these tasks have already been taught in preschool. We focus on family care tasks, which include putting items back in place after use, helping to set the table before meals, assisting in serving food, clearing the table after meals, washing dishes, washing fruit, throwing trash into the bin, sorting garbage, putting family members’ clothes into the laundry basket, hanging clothes and socks, sweeping, vacuuming, mopping, cleaning the bathroom or toilet, and helping family members carry or move items.

### 1.3. The Use of Technological Media

In preschools, teaching activities often employ picture books [18], thematic learning areas [19], teacher demonstrations, and practical life experiences to achieve effective learning outcomes. Today’s children, whether at home, in preschool institutions, or in public environments, frequently come into contact with technological products [20], such as Artificial Intelligence (AI), also known as machine intelligence. These diverse technologies also play an important role in informal education throughout individuals’ lives [21]. However, past research has suggested that when individuals are too young, exposure to technological products will have a negative impact on their brain and other development [22,23]. Consequently, traditional teaching models have dominated the design of preschool activities in the past. More recent research, however, has highlighted the potential of diverse learning activities, such as those provided through radio, television, and the internet, to promote information exchange, enhance interpersonal interaction, and foster knowledge innovation [24].

Mayer [25] proposed the “Cognitive Theory of Multimedia Learning” (CTML), which emphasizes how learners process information from multimedia materials, including images, audio, and text. The theory suggests that for meaningful learning to occur, learners must engage in a series of cognitive processes: first, selecting relevant verbal and visual information from the learning content; then, organizing this information into coherent mental representations; and finally, integrating these verbal and visual models into meaningful connections with long-term memory, thereby leading to a deeper understanding of the knowledge. Additionally, the theory introduces several multimedia design principles (including the Multimedia Principle) aimed at enhancing learning outcomes. Based on this theory, this study designs learning activities using concepts from various multimedia principles, such as stories, images, songs, and demonstrations to improve learning effectiveness.

Related research has also found that utilizing various technological media can help stimulate young children’s motivation for active learning [26] while connecting multiple perspectives in children’s learning processes, thereby enhancing learning outcomes [27]. Adams [28] noted that the appropriate use of technological media can improve cognitive and social skills as well as strengthen parent–teacher communication and relationships. Furthermore, Zhang [29] found that using computer-assisted storytelling helps children with delayed language development improve their verbal expression skills. Therefore, aligning with children’s developmental needs and incorporating technological diversity promotes the development of cognitive abilities, confidence, and related skills, while also fostering children’s intrinsic motivation to learn. This highlights the importance of effectively integrating technology into curricula [30]. Accordingly, this study utilizes AI robots to teach young children household tasks, aiming to understand the impact of AI robot-assisted instruction on children’s performance in housework.

### 1.4. Research Purpose

1. To examine how AI robot-assisted instruction in household tasks can enhance young children’s performance in housework;

2. To compare the effects of different teaching methods (AI robots, teachers, and no instruction) on young children’s performance in housework.

## 2. Methods

### 2.1. Research Design

This study adopted a quasi-experimental design, a method used when the random assignment of participants into experimental and control groups is not feasible within the research setting, thereby necessitating the use of existing groups (e.g., classes) for the experiment [31]. The experimental setting of this study was in preschools, where sampling was required to be conducted at the class level due to the preschools’ regulations. As a result, the ideal of “complete randomization” in sample assignment could not be achieved. However, to accommodate these practical constraints and better control the experiment’s content and external validity, this study employs a “nonequivalent pre-test–post-test control group design” to explore the differences in changes in household task abilities between the experimental group and two control groups before and after the intervention. The experimental design model of this study is presented in Table 1.

### 2.2. Sample

This study involved a total of 240 kindergarten children from preschools located in the Taipei metropolitan area (including Taipei City, New Taipei City, and Taoyuan City). Each group—the experimental group and two control groups—originally consisted of 80 participants; however, due to reasons such as illness preventing children from fully participating in the program, families relocating due to parents’ work, mid-study withdrawals, and incomplete questionnaires, the final valid sample size was reduced to 193. Among them, the experimental group had 65 participants, control group 1 had 53 participants, and control group 2 had 75 participants. The selected kindergartens followed a similar thematic activity-based curriculum design to minimize differences in teaching methods between the kindergartens.

### 2.3. Data Collection Process

Before conducting the AI robot- and teacher-led instructional activities, both the experimental group and the control groups underwent a pre-test to assess the children’s household task abilities prior to participating in the teaching activities. The experimental group and control group 2 engaged in teaching activities for three weeks, with sessions held twice a week, each lasting approximately 20–30 min. A post-test was administered within one week after the final teaching session to evaluate the outcomes following the use of different teaching methods. The data for both assessments were collected through online Google questionnaires, completed by the same primary caregiver (usually the mother) for each child. The responses were not anonymous to facilitate merging the data from the two assessments.

#### 2.3.1. Instrument

This study primarily adopted the “Survey on Children’s Preferences and Abilities in Household Chores” developed by Lee and Tang [17] to assess the household participation abilities of the experimental group and the two control groups during the pre-test and post-test. Since the main objective of this research is to explore the relationship between the use of AI robots and children’s performance in housework, both the pre-test and post-test included measurements of children’s performance in housework. The measurement methods for the relevant variables are described as follows:

##### Independent Variable—Group

The independent variable in this study is “group”, which refers to the different types of experimental treatments the children received. The participants were divided into three groups:

Experimental group “0” (children engaged in household learning activities led by an AI robot);

Control group 1 “1” (children did not receive any experimental treatment);

Control group 2 “2” (children engaged in household learning activities led by a teacher; although the activity content and name were identical to those of the experimental group, there was no involvement of the AI robot throughout the activity).

##### Outcome Variable—Children’s Housework Participation Ability (CHPA)

The outcome variable in this study is children’s housework participation ability, which was measured using items from the “Survey on Children’s Preferences and Abilities in Household Chores—Chores Related to Family Members” developed by Lee and Tang [17]. The original scale consists of 16 items but due to time constraints at the participating kindergarten, and after discussions with the teachers, the curriculum was designed to cover 12 household tasks that were more aligned with the children’s developmental needs. Therefore, the assessment was limited to these 12 tasks, which include the following: “putting items back in place after use”, “helping set the table before meals”, “helping serve food”, “helping clear the table after meals”, “washing dishes”, “washing fruit”, “throwing trash into the trash bin”, “sorting garbage”, “putting family members’ clothes into the laundry basket”, “hanging clothes and socks”, “sweeping, vacuuming, or mopping the floor”, “cleaning the bathroom or toilet”, and “helping family members carry or move items”. The scoring method ranges from 0 points (child did not perform the task) to 5 points (child completed the task independently). A higher score indicates better participation ability in that task, whereas a lower score reflects a lower level of participation ability.

#### 2.3.2. Use of the AI Robot

**Figure 1:**
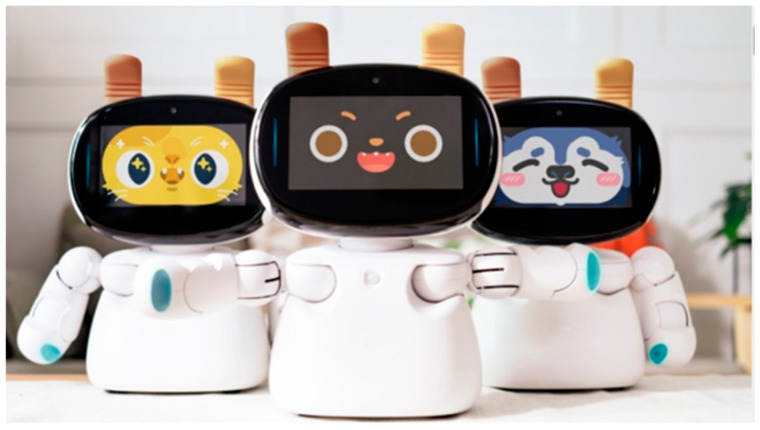
Kebbi Air S Robot.

In this study, the AI robot “Kebbi Air S” (Chinese name: 凱比) (Figure 1), developed by the “Nuwa Creation Team,” was chosen as the medium for facilitating children’s household task learning. Compared to other commercially available robots, Kebbi Air S was specifically designed for the children’s companionship and educational market. It excels in human–robot interaction, offering enhanced communication and interaction features. Additionally, Kebbi Air S is designed to initiate conversations and adapt interactions based on the user’s needs, making the human–robot interaction more natural. Therefore, this study utilized software (V2.0.82) and programming to integrate the robot into the preschool’s household task learning activities to support children’s performance in housework skills development.

In the preschool setting, the AI robot primarily served as a guide for children’s household task learning. The researcher utilized dedicated software tools such as the AI robot’s Professional Edition Programming Lab, Dialogue Training Room, and Content Editor to program the content for each household learning session. The course content was designed based on the children’s current developmental stage and was flexibly adjusted according to the on site conditions in the preschool.

**Figure 2:**
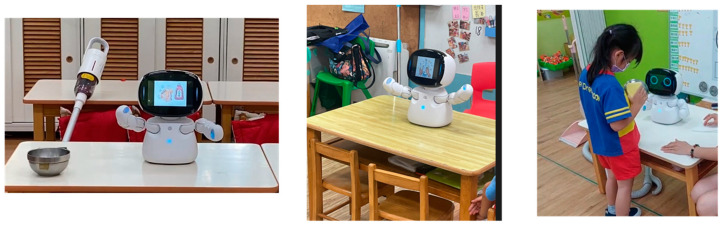
The AI robot demonstrates the dishwashing technique and encourages the children to engage in learning how to wash dishes.

Each time the AI robot entered the preschool to teach children household tasks, it began with engaging, playful music, language, and expressions to capture the children’s attention and immerse them in the learning environment. The AI robot’s teaching sessions in the preschool were designed to be lively and interactive. In addition to explaining how to perform household tasks, the robot encouraged the children, engaged them in discussions, allowed them to share their experiences, and incorporated singing and dancing as part of the learning process. The themes and procedures for the six sessions are outlined in Table 2 and Figure 2.

### 2.4. Research Ethics

Before this study commenced, approval was obtained from the Institutional Review Board (IRB) to ensure that the research adhered to international ethical principles, professional ethical standards, and relevant national laws. The researcher visited preschools that were willing to participate in the study, explaining the research objectives, implementation methods, expected outcomes, and ethical guidelines. Consent was obtained from both the preschools and the parents, with consent forms signed by both parties. To protect the rights of the participating children, all data collected during this study will be kept confidential and anonymized to safeguard the children’s privacy.

## 3. Results

### 3.1. Homogeneity Analysis of the Experimental Group, Control Group 1, and Control Group 2

According to Kuo [31], in quasi-experimental research, a pre-test must be conducted to assess the inequality between groups. To minimize factors that could affect internal validity, it is preferable that the extent of inequality between groups is as small as possible. One-way ANOVA was performed in this study to examine whether children’s “performance in housework” significantly differed based on their assigned “group”. The analysis revealed no significant differences in “performance in housework“ between the groups in the pre-test (F = 2.83, *p* > 0.05). This indicates that before the implementation of different teaching methods, the experimental group, control group 1, and control group 2 were homogeneous in terms of children’s household participation ability (Table 3).

### 3.2. The Analysis of Paired-Sample t-Tests

After confirming that the three groups were homogeneous, this study used paired-sample *t*-tests to examine whether there were significant differences in the children’s performance in housework before and after different household learning methods. The analysis showed that for the experimental group (which used AI robots for household learning activities), the pre-test mean (35.40) and the post-test mean (41.22) had a significant difference (*t* = 5.62, *p* < 0.001).

In contrast, for control group 1 (which did not receive any experimental treatment), there was no significant difference between the pre-test mean (31.75) and the post-test mean (31.30) (*t* = 0.32, *p* = 0.751). Additionally, for control group 2 (which received household learning instruction from a teacher), the pre-test mean (30.33) and the post-test mean (35.01) showed a significant difference (*t* = 4.97, *p* < 0.001).

In summary, it is evident that children’s “performance in housework” significantly improved after three consecutive weeks of household learning activities, both with AI robot intervention and with teacher-led instruction (Table 4).

### 3.3. Analysis of Differences in Housework Participation Ability Between the Experimental and Control Groups

The one-way ANOVA revealed that there were significant differences in children’s “performance in housework” after the implementation of different teaching methods (F = 8.65, *p* < 0.001). The Scheffe post hoc test showed that, after three consecutive weeks of household learning activities with the AI robot, the children’s performance in housework was significantly higher than those of children who either did not receive any instruction or were taught by a teacher (experimental group > control groups 1 and 2) (Table 5).

## 4. Discussion

The results of this study indicate that “AI robots teaching children household tasks” can enhance children’s performance in housework. Previous research on the use of AI robots with young children has found that interactions involving singing, dancing, simple conversations, and playing small games with robots help improve children’s attention during learning [32,33]. Studies on children with autism have also shown that robots can enhance their willingness to interact through pre-set scenarios, which leads to increased social interaction behaviors with the robot. Additionally, when robots are used in combination with picture cards and physical teaching aids during instructional activities, children are generally able to follow the robot’s guidance to complete learning tasks [34].

Furthermore, this study found that the teaching effectiveness of AI robots appears to be slightly better than that of teachers in instructing children in household tasks. The AI robot’s approach, incorporating storytelling, demonstrations, singing, and interactive dialogues, differs from traditional teacher-centered instruction. This aligns with the Cognitive Theory of Multimedia Learning [25], which emphasizes the positive impact of using diverse media on children’s learning. Therefore, it is recommended that preschools consider integrating technological media into children’s learning activities in the future. The use of AI robots could be a helpful tool in both general and special education settings, potentially reducing the burden on teachers while adding diversity to preschool teaching methods. Additionally, Weinberger et al. [27] suggested that the use of technological media can enhance children’s learning motivation. Whether using AI robots to teach household tasks can increase children’s motivation and willingness to participate in household chores—and thus improve their participation abilities—requires further investigation in future studies.

During the course of this study, it was observed that using the robot in the lessons easily sparked children’s curiosity and enjoyment; however, during the process, it was also noted that the small screen size of the Kebbi Air S robot used in this study made it difficult for children seated at the back to see clearly, leading to pushing and shoving as they tried to move forward or causing some children to struggle with understanding the household task techniques in a timely manner. This, in turn, partially affected the learning outcomes. Therefore, it is recommended that in future teaching sessions, the use of a projector along with adult guidance would be beneficial to ensure that all children can grasp the learning content promptly.

## Figures and Tables

**Table 1:** Experimental design for enhancing preschoolers’ performance in housework.

	Pre-Test	Experimental Treatment	Post-Test
Experimental group (*n* = 65)	T1	X	T2
Control group 1 (*n* = 53)	C11		C21
Control group 2 (*n* = 75)	C12		C22

**Table 2:** Kebbi Air S robot’s intervention in children’s housework learning activities list.

Number of Activities	Activity Title	Activity Procedure
No. 1	What are the household chores?	Through storytelling by the AI robot, children were introduced to the concept of household chores, which helped increase their interest in participating in these tasks.
No. 2	I can tidy up now.	The AI robot used pictures for explanations, employed physical teaching aids for children to demonstrate on stage, and incorporated singing and dancing to related songs about tidying up, helping the children learn how to organize and put away items.
No. 3	I can set the table and serve dishes.	The AI robot engaged in conversations with the children, encouraging them to share their experiences of performing household chores over the past few days. It used pictures for explanations and reminders, and after the children practiced using physical teaching aids on stage, the robot provided verbal praise. Additionally, the robot sang songs with the children to help them develop the ability to prepare for mealtime.
No. 4	After meals, I can clean the dishes and the floor.	The AI robot encouraged the children to share their recent experiences with household chores and provided verbal praise. It then used pictures for explanations and employed physical teaching aids to allow the children to practice proper techniques for cleaning dishes and the floor on stage. The robot also reminded the children to help with cleaning up after meals at home.
No. 5	I can organize the laundry.	After the children shared their experiences of performing household chores in the past few days, the AI robot used singing and dancing to guide them into the learning environment. The AI robot then enhanced the children’s ability to organize clothing by combining picture explanations with question-and-answer sessions and having the children practice through hands-on demonstrations on stage.
No. 6	I can help and take care of my family.	The AI robot first informed the children that this would be the final session of teaching household tasks at the preschool and gave positive praise for their dedicated learning throughout the previous sessions. It then used pictures for explanations and reminders to help the children learn how to assist in caring for their family members. Through singing and dancing, the AI robot encouraged the children to continue performing household chores with their families at home.

**Table 3:** Homogeneity analysis of the experimental group, control group 1, and control group 2.

	Groups	(*n*)	(M)	(SD)	B W T	df	SS	MS	F	*p*
Children’s housework participation ability—pre-test	0: experimental group	65	35.4	13.7	B	2	927.2	463.6	2.83	0.061
	1: control group 1	53	31.8	12.0	W	190	31,096.1	163.7		
	2: control group 2	75	30.3	12.5	T	192	32,023.3			

*p* > 0.05.

**Table 4:** The analysis of paired-sample *t*-tests.

Group	Pre-Test		Post-Test		*t*	*p*
	M	SD	M	SD		
Experimental group	35.40	13.74	41.22	12.92	5.62 ***	0.000
Control group 1	31.75	12.02	31.30	14.80	0.32	0.751
Control group 2	30.33	12.47	35.01	12.18	4.97 ***	0.000

*** *p* < 0.001.

**Table 5:** Analysis of differences in performance in housework between the experimental and control groups.

	Groups	(*n*)	(M)	(SD)	B W T	df	SS	MS	F	*p*	Scheffe
Children’s housework participation ability—post-test	0: experimental group	65	41.2	12.9	B	2	3009.5	1504.8	8.65 ***	0.000	experimental group > control group 1 experimental group > control group 2
	1: control group 1	53	31.3	14.8	W	190	33,057.1	174.0			
	2: control group 2	75	35.0	12.2	T	192	36,066.7				

*** *p* < 0.001.

## Data Availability

The data presented in this study are available on request from the first author. The data are not publicly available due to privacy and ethical reasons.
